# Variability and Randomness of the Instantaneous Firing Rate

**DOI:** 10.3389/fncom.2021.620410

**Published:** 2021-06-07

**Authors:** Rimjhim Tomar, Lubomir Kostal

**Affiliations:** ^1^Department of Computational Neuroscience, Institute of Physiology, Czech Academy of Sciences, Prague, Czechia; ^2^Second Medical Faculty, Charles University, Prague, Czechia

**Keywords:** variability, randomness, firing rate, entropy, rate coding, neural coding, temporal coding, instantaneous firing rate

## Abstract

The apparent stochastic nature of neuronal activity significantly affects the reliability of neuronal coding. To quantify the encountered fluctuations, both in neural data and simulations, the notions of variability and randomness of inter-spike intervals have been proposed and studied. In this article we focus on the concept of the instantaneous firing rate, which is also based on the spike timing. We use several classical statistical models of neuronal activity and we study the corresponding probability distributions of the instantaneous firing rate. To characterize the firing rate variability and randomness under different spiking regimes, we use different indices of statistical dispersion. We find that the relationship between the variability of interspike intervals and the instantaneous firing rate is not straightforward in general. Counter-intuitively, an increase in the randomness (based on entropy) of spike times may either decrease or increase the randomness of instantaneous firing rate, in dependence on the neuronal firing model. Finally, we apply our methods to experimental data, establishing that instantaneous rate analysis can indeed provide additional information about the spiking activity.

## 1. Introduction

One of the primary research areas of computational neuroscience is dedicated to understanding the principles of neuronal coding, i.e., the way information is embedded into neuronal signals. It is generally understood that neurons use brief electrical impulses (called action potentials or spikes) to convey information. The way information is presented in the time sequence of spikes, however, is still a matter of debate (Shadlen and Newsome, 1994; Stein et al., 2005).

A widely accepted answer to the problem is the rate coding hypothesis, which says that the neurons transmit information through the average number of spikes sent along the axon per a certain time window (this is called the *mean firing rate*). The origin of this theory is credited to Edgar Adrian who found out that the firing rate of the stretch receptor of a frog's muscle changes as a function of stimuli (Adrian, 1926). However, since then, many studies have shown that neurons can encode information without necessarily changing the mean firing rate in response to a stimulus (Perkel and Bullock, 1968; Gerstner and Kistler, 2002; Rigotti et al., 2013; Dettner et al., 2016) prompting the temporal coding hypothesis, which states that the temporal structure of the ISIs is also employed in the embedding of neural information in the spike train (Theunissen and Miller, 1995). Theoretically, information can be encoded in the temporal pattern of the ISIs in an infinite number of ways (Thorpe and Gautrais, 1997; Jacobs et al., 2009; Ainsworth et al., 2012), therefore measures are needed to quantify the features of spiking neuronal signals from different perspectives (Perkel and Bullock, 1968; Victor and Purpura, 1997; Buračas and Albright, 1999; Rieke et al., 1999; Nemenman et al., 2004). A possible way of looking at the role of temporal structures can be through variability coding hypothesis (Perkel and Bullock, 1968) which states that neuronal variability may not be entirely noise, and part of it might reflect the aspects of neural code that is not yet understood; whether it is the variability of the ISIs or of the firing rate. A standard measure for comparing variability in spike trains is through the coefficient of variation (*C*_*V*_) which is defined as the ratio of standard deviation to the mean of ISIs (Barbieri and Brunel, 2007). Variability of the firing rate is measured by the Fano factor (Ditlevsen and Lansky, 2011; Rajdl and Lansky, 2013; Stevenson, 2016) which is defined as the ratio of variance to the mean number of spikes in a fixed time window.

Another concept that is similar to variability but not equivalent, is the notion of randomness (Kostal et al., 2007b). Variability and randomness both are used as a differentiating measure in the cases where spike trains might seem similar from the rate coding perspective. It is important to distinguish between the two quantities because highly variable data might not be random at all if it consists of relatively predictable values. For example multi-modal data with well separated peaks may have higher variability than uniformly distributed data where the outcomes are the least predictable. Shannon's entropy (Shannon and Weaver, 1949) is widely used to measure randomness (Steuer et al., 2001; McDonnell et al., 2011; Watters and Reeke, 2014), however it is not suitable for continuous distributions. Few other randomness measures based on entropy have been used in neural context recently. In Kostal et al. (2007a) and Kostal et al. (2013) the authors propose a randomness measure for ISIs, creating an alternative to *C*_*V*_; whereas an entropy based randomness measure of spike counts is introduced in Rajdl et al. (2017), analogous to the Fano factor.

The instantaneous rate is often ambiguously defined as the inverse of a certain inter-spike interval, e.g., of the first complete ISI after stimulus onset, or of a combination of first *n* intervals, etc. (Bessou et al., 1968; Awiszus, 1988; Lansky et al., 2004). However, statistically consistent definition of the instantaneous rate (Kostal et al., 2018) cannot depend on the “intrinsic” timing given by the particular spike train realization (e.g., the first evoked spike). Instead, it must be evaluated with respect to the “external” time frame, consistently across trials, i.e., asynchronously with respect to individual spike train realizations. In this paper we consider models of spike trains described by renewal processes, and investigate the dependence between the ISI dispersion coefficients and instantaneous rate dispersion coefficients and emphasize that instantaneous rate provides another perspective in the evaluation of neuronal data.

The paper is divided as follows: first, the concept of instantaneous rate is introduced. Next, the concepts of variability and randomness are defined formally. In section 2, we derive the instantaneous rate distribution and the related dispersion measures for a few of the most commonly used models of steady-state neuronal activity. Included models are restricted to the framework of renewal spiking activity for the purpose of this paper. In section 3, we compare the dispersion characteristics of the neuronal models from rate coding and temporal coding perspectives. Upon comparison we find that for some neuronal models, the firing patterns have a different level of randomness in different settings whereas for others, the changed perspective of observation (from rate to temporal) does not affect the randomness of the data. More details on this is available in section 4.

## 2. Materials and Methods

### 2.1. Instantaneous Rate

**Figure 1:**
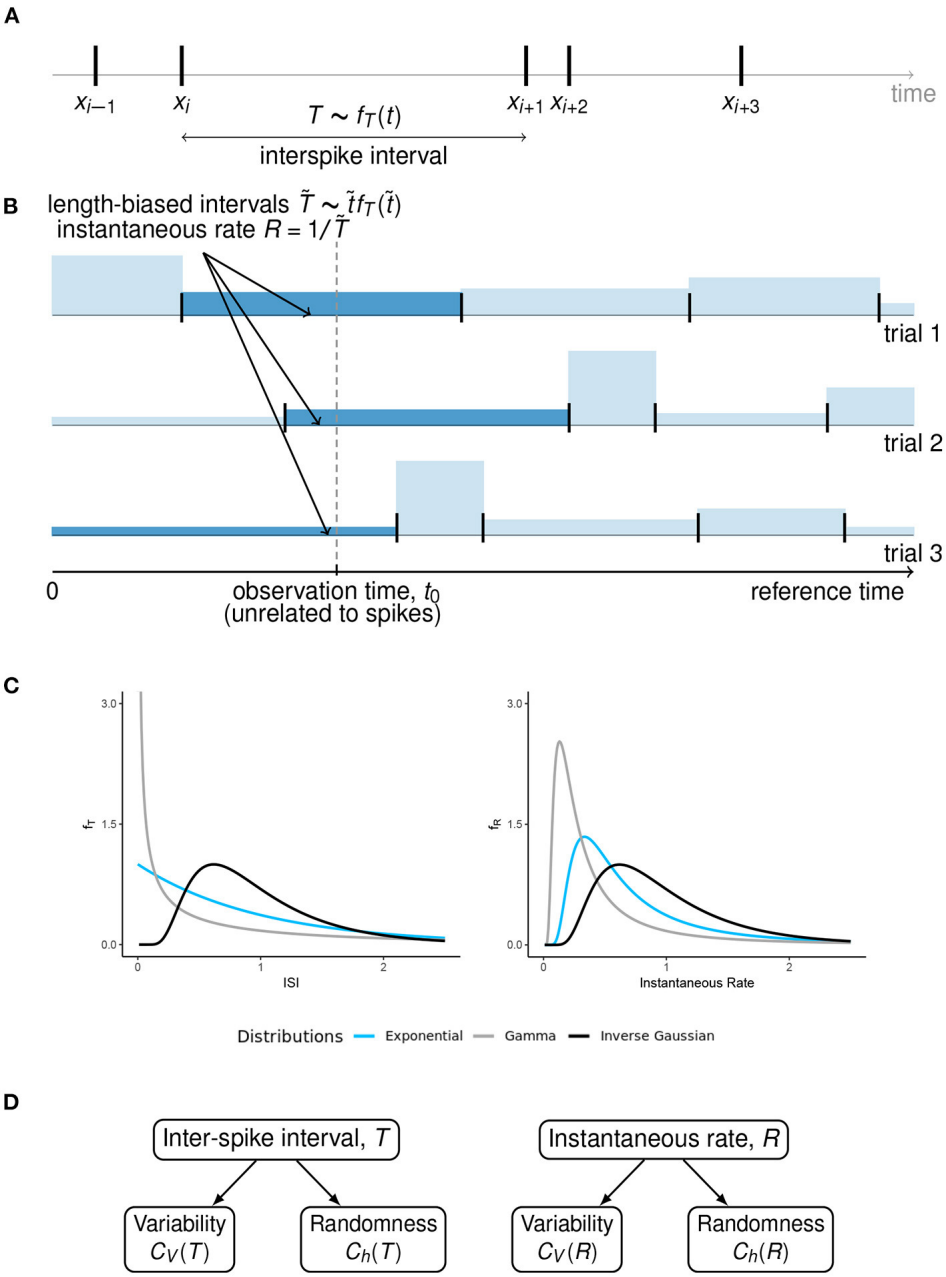
Length-biased ISIs and instantaneous rate. **(A)** An overview of the ISIs when the spikes occur at times *X*_*i*−1_, *X*_*i*_, *X*_*i*+1_, …. We assume that the ISIs are independent and identically distributed with pdf *f*_*T*_(*t*), under steady state conditions. **(B)** When the observation time *t*_0_ is fixed with respect to some reference time, unrelated to the spike times, the probability of observing a particular ISI is proportional to its length. These “length-biased” intervals ($\tilde{T}$) are used to define the instantaneous rate *R* with the property 𝔼(*R*) = 1/𝔼(*T*). **(C)** A graphical representation of how the ISI distributions can visually differ from the instantaneous rate distributions, for some well-known ISI models with equal mean firing rate. **(D)** A summary of the concepts of variability and randomness for the two ways of spike train description that we have considered in this article.

The class of stochastic processes whose realization consists of a sequence of point events in time is called *stochastic point processes* (Cox and Miller, 1965). Neuronal spike trains are often described as stochastic point processes, with spikes corresponding to events. There are essentially two ways of describing point processes, either in terms of the number of events occurring in a time window, or in terms of the intervals between these events. Consequently, a spike train can be described either by using a sequence of the occurrence times of its individual spikes, *X*_1_, *X*_2_, … (see Figure 1A) or through the ISIs defined as *T*_*i*_ = *X*_*i*+1_ − *X*_*i*_. Generally, the point process is *stationary* if the underlying joint probability distribution of the numbers of the spikes in *k* time intervals $\left(t_{1}^{\prime }+h,t_{1}^{\prime \prime }+h\right),\left(t_{2}^{\prime }+h,t_{2}^{\prime \prime }+h\right),\ldots \left(t_{k}^{\prime }+h,t_{k}^{\prime \prime }+h\right)$ does not depend on displacement variable *h* (Cox and Lewis, 1966, p. 59). In this paper, we consider an important class of stationary point processes, the renewal point processes, in which the length *T* of consequent ISIs is an independently and identically distributed random variable with the probability distribution function (pdf) *f*_*T*_, *T* ~ *f*_*T*_(*t*). Renewal processes are often used to model the activity of spontaneously active cells (Tuckwell, 1988).

Let the number of spikes that occur in the time interval [0, *w*] be denoted by *N*(*w*). A natural way of calculating the firing rate is to divide the number of spikes *N*(*w*) by the time window *w*. The mean of ISIs, 𝔼(*T*), satisfies the following relationship with the mean of the counting process 𝔼[*N*(*w*)] (Cox and Lewis, 1966; Rudd and Brown, 1997),

(1) $$\begin{array}{l} \underset{w\to \infty }{\lim}\frac{𝔼\left(N\left(w\right)\right)}{w}=\frac{1}{𝔼\left(T\right)}=\lambda . \end{array}$$

where λ is the point process intensity (Cox and Lewis, 1966).

For finite *w*, Equation (1) holds true for renewal processes only if time origin *t*_0_ is arbitrary, i.e., it is not related to the renewal point process realization (Figure 1B). In this case, *t*_0_ falls into some ISI, say *T*_*k*_. Then the sequence of random variables (*W, T*_*k*+1_, *T*_*k*+2_, …), where *W* is the time to the first spike from the origin, is not stationary. Moreover, the point process intensity λ is equal to the mean firing rate. The corresponding renewal process is referred to as *equilibrium renewal process*, as opposed to the *ordinary renewal process* which starts from an arbitrary spiking event and all the ISIs follow the same renewal pdf (Cox and Miller, 1965).

The instantaneous firing rate is typically defined as the inverse of the ISIs (1/*T*) (Pauluis and Baker, 2000). However, as proven in Lansky et al. (2004), the mean instantaneous firing rate is higher than or equal to the mean firing rate,

(2) $$\begin{array}{l} 𝔼\left(\frac{1}{T}\right)\geq \frac{1}{𝔼\left(T\right)} \end{array}$$

with equality if all the ISIs are of the same length.

The undesirable inequality in Equation (2) becomes equality once we realize that the “time instant” (at which the instantaneous rate is measured) does not generally coincide with a spike. As shown in Kostal et al. (2018), the instant is chosen with respect to the “external” time frame, across trials, i.e., asynchronously with respect to individual spike train realizations. Consequently, the probabilities of observed ISIs ($\tilde{T}$) are proportional to their length, $\tilde{T}\sim \lambda \tilde{t}f_{T}\left(\tilde{t}\right)$ (Figure 1B). The mean inverse of these length-biased ISIs equals to the mean firing rate λ.

The instantaneous rate $R=1/\tilde{T}$ observed, in this case, is a random variable described by the pdf,

(3) $$\begin{array}{l} f_{R}\left(r\right)=\frac{1}{𝔼\left(T\right)}\frac{f_{T}\left(1/r\right)}{r^{3}}, \end{array}$$

For a detailed overview of the derivations, refer to Kostal et al. (2018).

An immediate consequence of Equation (3) is,

(4) $$\begin{array}{l} 𝔼\left(\frac{1}{\tilde{T}}\right)=𝔼\left(R\right)=\frac{1}{𝔼\left(T\right)}=\lambda . \end{array}$$

Hence, for the purpose of the derivation of instantaneous rate distributions for different models of steady-state firing (Figure 1C), we will restrict ourselves to the case of equilibrium renewal process. The variability of instantaneous rate is explored in the next section.

### 2.2. Quantification of Variability and Randomness

The most common method to measure statistical dispersion of ISIs, described by a continuous positive random variable *T*, is the standard deviation σ(*T*), defined as

(5) $$\begin{array}{l} \sigma \left(T\right)=\sqrt{𝔼\left({\left[T-𝔼\left(T\right)\right]}^{2}\right)}. \end{array}$$

The *relative* dispersion measure quantity is known as coefficient of variation *C*_*V*_(*T*),

(6) $$\begin{array}{l} C_{V}\left(T\right)=\lambda \sigma \left(T\right) \end{array}$$

where λ = 1/𝔼(*T*). The coefficient of variation *C*_*V*_(*T*) is a dimensionless quantity and its value does not depend on the choice of the units of ISIs or on linear scaling; hence it can be used to meaningfully compare the ISI distributions with different means, unlike σ(*T*) (Softky and Koch, 1993; Doron et al., 2014).

From Equation (3), we can write the standard deviation for the instantaneous rate as

(7) $$\begin{array}{l} \sigma \left(R\right)=\sqrt{\lambda 𝔼\left(1/T\right)-\lambda^{2}}, \end{array}$$

which leads to the relative dispersion measure,

(8) $$\begin{array}{l} C_{V}\left(R\right)=\sqrt{\frac{𝔼\left(1/T\right)}{\lambda }-1}. \end{array}$$

From Equation (5), it follows that σ or *C*_*V*_ measure how much the probability distribution is “spread” with respect to the mean value but these quantities do not describe all possible differences between spike trains with equal rate coding characteristics. Spike trains of equal variability may still differ in higher than second statistical moments. Moreover, neither σ nor *C*_*V*_ quantifies how random or unpredictable the outcomes are Kostal et al. (2007a).

To quantify randomness as a further describing characteristic of a spike train, entropy based measures like differential entropy (Shannon and Weaver, 1949), *h*, have been proposed

(9) $$\begin{array}{l} h\left(f_{X}\right)=-\int f_{X}\left(x\right)\ln\;f_{X}\left(x\right)\text{d}x. \end{array}$$

where *X* is a continuous random variable with pdf *f*_*X*_. However, *h*(*f*_*X*_) by itself can not be used as a measure of randomness since it can take both positive and negative values and depends on the scaling of the random variable *X*. Kostal and Marsalek (2010) proposed the entropy-based dispersion coefficient σ_*h*_,

(10) $$\begin{array}{l} \sigma_{h}=\exp\left(h\left(f_{X}\right)-1\right). \end{array}$$

Entropy-based dispersion σ_*h*_ can be interpreted with the help of asymptotic equipartition property (Principe, 2010; Cover and Thomas, 2012), the details of which can be found in Kostal et al. (2013). Analogous to Equation (6), the relative entropy-based measure of dispersion, *C*_*h*_ is defined as

(11) $$\begin{array}{l} C_{h}=\lambda \sigma_{h}. \end{array}$$

An immediate consequence of the above equation is that the maximum value of *C*_*h*_ is *C*_*h*_ = 1, which occurs only in the case of exponential *f*_*T*_.

## 3. Results

Among the different point process models of stationary neuronal activity, we have chosen the classically used Gamma, lognormal, inverse Gaussian, shifted exponential, and the mixture of exponential distributions (Tuckwell, 1988).

First three distributions are part of the scale family (Casella and Berger, 2002), i.e., their ISI pdfs *f*_*T*_(*t*; λ), explicitly parameterized by the intensity λ, satisfy the relationship

(12) $$\begin{array}{l} f_{T}\left(t,\lambda \right)=\lambda f_{T}\left(t\lambda ,1\right). \end{array}$$

We explore these neuronal models in the following subsections (Figure 1D). Detailed derivations of the following results are provided in the Supplementary Material for better legibility.

### 3.1. Gamma Distribution

Gamma distribution is frequently used as a descriptor of ISIs in experimental data analysis (Levine, 1991; McKeegan, 2002; Reeke and Coop, 2004; Pouzat and Chaffiol, 2009), its pdf is

(13) $$\begin{array}{l} f_{T}\left(t\right)=\frac{b^{a}t^{a-1}e^{-bt}}{\Gamma \left(a\right)}, \end{array}$$

where $\Gamma \left(z\right)=\int_{0}^{\infty }x^{z-1}e^{-x}\text{d}x$ is the gamma function and *a* > 0, *b* > 0 are the parameters. The mean firing rate and the coefficient of variation are,

(14) $$\begin{array}{l} \lambda =\frac{b}{a},\;C_{V}\left(T\right)=\frac{1}{\sqrt{a}}. \end{array}$$

Using Equation (9), the expression for the entropy of the ISI distribution is derived as

(15) $$\begin{array}{l} h\left(f_{T}\right)=\log\left(\frac{\Gamma \left(a\right)}{b}e^{a+\left(1-a\right)\psi \left(a\right)}\right), \end{array}$$

where ψ(*x*) = Γ′(*x*)/Γ(*x*) is the digamma function.

Substituting the values from Equations (14) and (15) into Equation (11), gives the dispersion coefficient of randomness,

(16) $$\begin{array}{l} C_{h}\left(T\right)=\frac{\Gamma \left(a\right)}{a}e^{a+\left(1-a\right)\psi \left(a\right)-1}. \end{array}$$

The instantaneous rate distribution *f*_*R*_(*r*) is obtained through Equation (3) and it follows the inverted gamma distribution,

(17) $$\begin{array}{l} f_{R}\left(r\right)=\frac{b^{a+1}r^{-a-2}e^{-b/r}}{\Gamma \left(a+1\right)}. \end{array}$$

Coefficient of variation *C*_*V*_(*R*) is evaluated through Equation (8),

(18) $$\begin{array}{l} C_{V}\left(R\right)=\sqrt{\frac{1}{a-1}},\;a>1. \end{array}$$

The differential entropy for *f*_*R*_(*r*) is,

(19) $$\begin{array}{l} h\left(f_{R}\right)=\log\left(\Gamma \left(a+1\right)be^{\left(a+1\right)-\left(a+2\right)\psi \left(a+1\right)}\right), \end{array}$$

and the dispersion coefficient of randomness is,

(20) $$\begin{array}{l} C_{h}\left(R\right)=a\Gamma \left(a+1\right)e^{a-\left(a+2\right)\psi \left(a+1\right)}. \end{array}$$

The dispersion measures *C*_*V*_(*T*) and *C*_*V*_(*R*) are related through the following equation,

(21) $$\begin{array}{l} C_{V}\left(R\right)=\frac{C_{V}\left(T\right)}{\sqrt{1-C_{V}{\left(T\right)}^{2}}}. \end{array}$$

**Figure 2:**
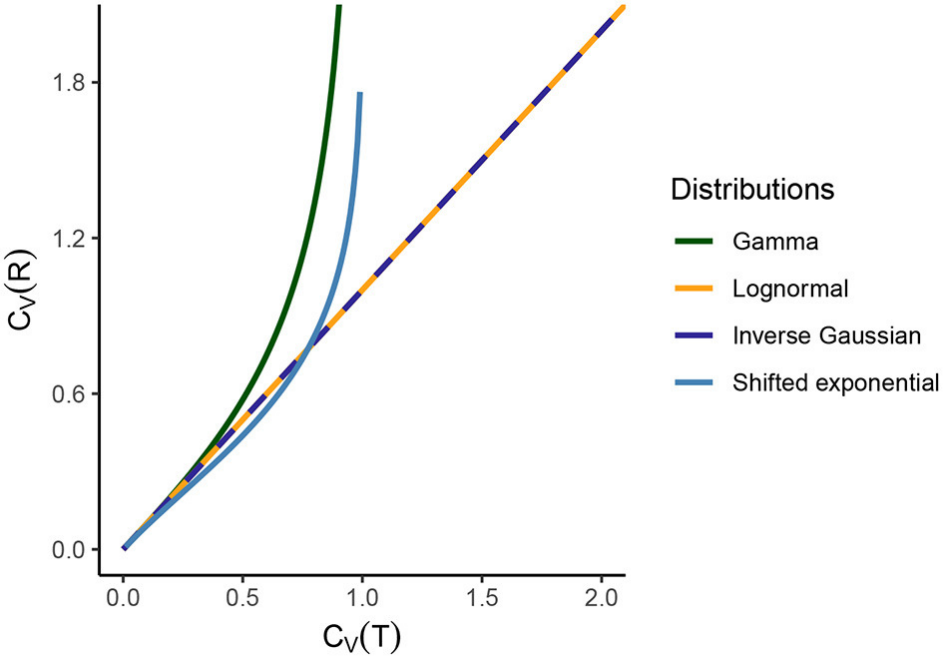
Comparison of ***C*_*V*_(*T*)** and ***C*_*V*_(*R*)** for several standard statistical ISI models. For the gamma distribution, *C*_*V*_(*R*) → ∞ as *C*_*V*_(*T*) → 1, since gamma distribution becomes exponential for *C*_*V*_(*T*) = 1. For lognormal and inverse Gaussian distribution, the relationship between the dispersion coefficients is an identity. For shifted exponential distribution, *C*_*V*_(*T*) and *C*_*V*_(*R*) depend on the mean firing rate λ and the refractory period τ. Hence, we vary the value of *C*_*V*_(*T*) and *C*_*V*_(*R*) (consequently of λ and τ) and we see that *C*_*V*_(*R*) > *C*_*V*_(*T*) until *C*_*V*_(*T*) = 0.7715 but then instantaneous rate distribution maintains a higher variability than ISIs. As *C*_*V*_(*T*) → 1, the shifted exponential becomes exactly exponential and therefore *C*_*V*_(*R*) → ∞.

This relationship is illustrated in Figure 2 and we see that *C*_*V*_(*R*) → ∞ as *C*_*V*_(*T*) → 1. For *C*_*V*_(*T*) = 1, gamma distribution becomes exponential distribution with pdf,

(22) $$\begin{array}{l} f_{T}\left(t\right)=\lambda e^{-\lambda t}. \end{array}$$

Note that the firing intensity λ completely characterizes the exponential distribution and that *C*_*V*_(*T*) = 1, regardless of the value of λ. The entropy *h*(*f*_*T*_) of the exponential distribution is

(23) $$\begin{array}{l} h\left(f_{T}\right)=1-\ln\;\lambda . \end{array}$$

One of the key features of the exponential distribution is that, for a fixed mean firing rate λ, it maximizes the differential entropy among all probability distributions on the real positive half line (Cover and Thomas, 2012). Deriving from Equation (11),

(24) $$\begin{array}{l} C_{h}\left(T\right)=1. \end{array}$$

**Figure 3:**
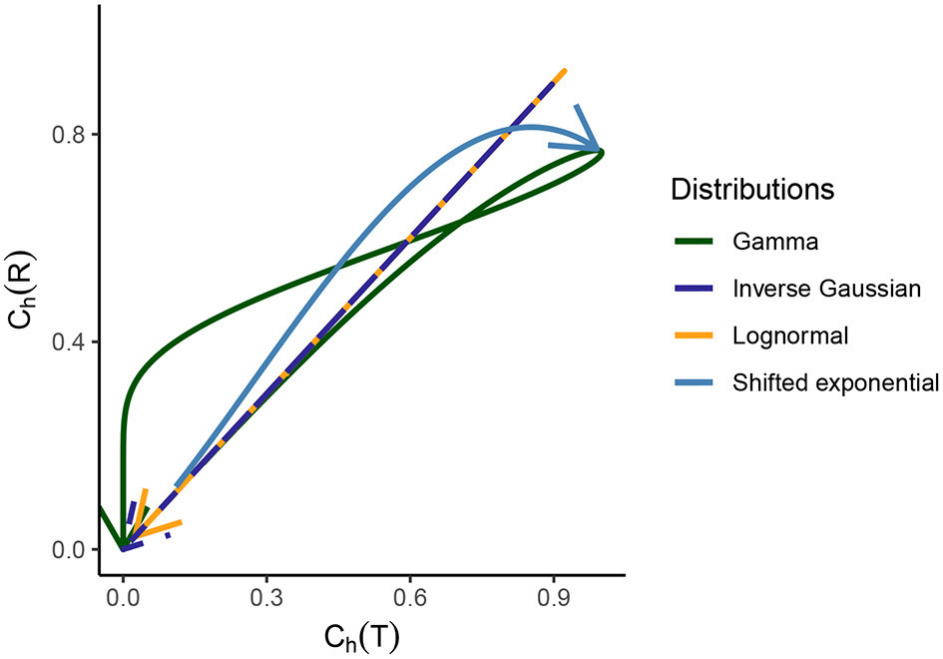
Relationship between the dispersion coefficients of randomness for the gamma, lognormal, inverse Gaussian, and shifted exponential distribution of ISIs. Starting from the origin, the arrow indicated to the end corresponds to *C*_*V*_(*T*) values ranging from 0 to ∞. For the first three distributions, which are part of the scale family and for which we consider λ = 1 for a meaningful comparison, it holds that for *C*_*V*_(*T*) → 0, we see that *C*_*h*_(*T*) → 0 for *C*_*h*_(*R*) → 0. As *C*_*V*_(*T*) grows, the randomness grows for ISIs and instantaneous rate in the beginning. After a certain point, the randomness starts to decline and as *C*_*V*_(*T*) → ∞, we have *C*_*h*_(*T*) → 0 and *C*_*h*_(*R*) → 0. For the shifted exponential distribution where we have to consider a varying *C*_*V*_(*T*) and for that a varying mean firing rate λ and refractory period τ, *C*_*h*_(*T*) increases monotonously as a function of *C*_*V*_(*T*), whereas *C*_*h*_(*R*) obtains its maximum for *C*_*V*_(*T*) = 0.85 and then declines.

The corresponding instantaneous rate distribution, follows from Equation (3),

(25) $$\begin{array}{l} f_{R}\left(r\right)=\frac{\lambda^{2}e^{-\lambda /r}}{r^{3}} \end{array}$$

which is the inverse gamma distribution mentioned in Equation (17) with *a* = 1 and *b* = λ. For the inverse gamma distribution, the second moment exists only when *a* > 1. In Figure 3, we can see where gamma distribution becomes exponential and has *C*_*h*_(*T*) = 1.

### 3.2. Lognormal Distribution

The lognormal distribution represents a common ISI descriptor in experimental data analysis (Levine, 1991; Pouzat and Chaffiol, 2009), even though it is rarely presented as a result of any of the neuronal models (Bershadskii et al., 2001). The pdf is,

(26) $$\begin{array}{l} f_{T}\left(t\right)=\frac{1}{\sigma t\sqrt{2\pi }}\text{exp}\left\{-\frac{{\left(\ln\;t-\ln\;m\right)}^{2}}{2\sigma^{2}}\right\}, \end{array}$$

where *m* is the scale parameter and σ > 0 is the shape parameter. The mean firing rate and coefficient of variation are as follows,

(27) $$\begin{array}{l} \lambda =\frac{1}{me^{\sigma^{2}/2}},\;C_{V}\left(T\right)=\sqrt{e^{\sigma^{2}}-1}. \end{array}$$

We compute the differential entropy

(28) $$\begin{array}{l} h\left(f_{T}\right)=\frac{1}{2}\log\left(2\pi e\sigma^{2}m^{2}\right), \end{array}$$

and the dispersion coefficient of randomness,

(29) $$\begin{array}{l} C_{h}\left(T\right)=\sigma \sqrt{2\pi }e^{-\left(\sigma^{2}+1\right)/2}. \end{array}$$

The instantaneous rate distribution follows the pdf,

(30) $$\begin{array}{l} f_{R}\left(r\right)=\frac{1}{me^{\sigma^{2}/2}}\frac{1}{r^{2}\sigma \sqrt{2\pi }}\exp\left\{-\frac{{\left(\ln\;r+\ln\;m\right)}^{2}}{2\sigma^{2}}\right\}, \end{array}$$

with

(31) $$\begin{array}{l} C_{V}\left(R\right)=\sqrt{e^{\sigma^{2}}-1}. \end{array}$$

The expression for the differential entropy is derived from Equation (11),

(32) $$\begin{array}{l} h\left(f_{R}\right)=\frac{1}{2}\log\left(\frac{2\pi e\sigma^{2}}{m^{2}e^{2\sigma^{2}}}\right). \end{array}$$

The dispersion coefficient is evaluated as,

(33) $$\begin{array}{l} C_{h}\left(R\right)=\sigma \sqrt{2\pi }e^{-\left(\sigma^{2}+1\right)/2}. \end{array}$$

For the lognormal distribution, there is a “symmetric” relationship between *f*_*T*_(*t*) and *f*_*R*_(*r*) (Kostal et al., 2018),

(34) $$\begin{array}{l} f_{R}\left(r;\lambda \right)=f_{T}\left(r;1/\lambda \right), \end{array}$$

i.e., the shape of the probability distributions of ISI and instantaneous rates are exactly the same for λ = 1. Furthermore, the relationship between *C*_*V*_(*T*) and *C*_*V*_(*R*) is that of an identity (Figure 2),

(35) $$\begin{array}{l} C_{V}\left(R\right)=C_{V}\left(T\right) \end{array}$$

and from Equations (29) and (33), we have

(36) $$\begin{array}{l} C_{h}\left(T\right)=C_{h}\left(R\right). \end{array}$$

For lognormal distribution, the randomness and variability of instantaneous rate and ISI are the same, regardless of the perspective, as seen in Figures 2, 3.

### 3.3. Inverse Gaussian Distribution

Inverse Gaussian distribution is often fitted to experimentally observed ISIs (Gerstein and Mandelbrot, 1964; Berger et al., 1990; Levine, 1991; Pouzat and Chaffiol, 2009). The time that a Weiner process with positive drift takes to reach the first passage time is distributed according to the inverse Gaussian distribution. The probability density of inverse Gaussian distribution can be expressed as a function of its mean *a* > 0 and scale parameter *b* > 0

(37) $$\begin{array}{l} f_{T}\left(t\right)=\sqrt{\frac{a}{2\pi bt^{3}}}\text{exp}\left\{-\frac{1}{2b}\frac{{\left(t-a\right)}^{2}}{at}\right\}, \end{array}$$

with

(38) $$\begin{array}{l} \lambda =\frac{1}{a},\;C_{V}\left(T\right)=\sqrt{b}. \end{array}$$

From Equation (9), the expression for differential entropy is,

(39) $$\begin{array}{l} h\left(f_{T}\right)=\frac{1}{2}\log\left(2\pi a^{2}be\right)+\frac{3e^{1/b}}{\sqrt{2\pi b}}K^{\left(1,0\right)}\left(-\frac{1}{2},\frac{1}{b}\right), \end{array}$$

where $K_{\nu }^{\left(1,0\right)}\left(z\right)$ is the derivative of the modified Bessel function of the second kind (Abramowitz and Stegun, 1972).

(40) $$\begin{array}{l} K_{\nu }^{\left(1,0\right)}\left(z\right)=\frac{\partial }{\partial \nu }K_{\nu }\left(z\right). \end{array}$$

Equation (11) gives,

(41) $$\begin{array}{l} C_{h}\left(T\right)=\sqrt{\frac{2\pi b}{e}}\exp\left\{\frac{3e^{1/b}}{\sqrt{2\pi b}}K^{\left(1,0\right)}\left(-\frac{1}{2},\frac{1}{b}\right)\right\}. \end{array}$$

The instantaneous firing rate follows the distribution

(42) $$\begin{array}{l} f_{R}\left(r\right)=\sqrt{\frac{1}{2\pi abr^{3}}}\exp\left\{-\frac{1}{2b}\frac{{\left(1-ar\right)}^{2}}{ar}\right\}. \end{array}$$

From Equation (8),

(43) $$\begin{array}{l} C_{V}\left(R\right)=\sqrt{b}, \end{array}$$

and from Equation (9), differential entropy is,

(44) $$\begin{array}{l} h\left(f_{R}\right)=\frac{1}{2}\log\left(\frac{2\pi be}{a^{2}}\right)+\frac{3e^{1/b}}{\sqrt{2\pi b}}K^{\left(1,0\right)}\left(-\frac{1}{2},\frac{1}{b}\right). \end{array}$$

The expression for the dispersion coefficient of randomness is as follows,

(45) $$\begin{array}{l} C_{h}\left(R\right)=\sqrt{\frac{2\pi b}{e}}\exp\left\{\frac{3e^{1/b}}{\sqrt{2\pi b}}K^{\left(1,0\right)}\left(-\frac{1}{2},\frac{1}{b}\right)\right\}. \end{array}$$

Analogous to the lognormal distribution, we observe that the inverse Gaussian distribution also satisfies the “symmetrical” property (Equation 34) and,

(46) $$\begin{array}{l} C_{V}\left(T\right)=C_{V}\left(R\right),\;C_{h}\left(T\right)=C_{h}\left(R\right). \end{array}$$

Results from Figures 2, 3 illustrate this curious property that the randomness and variability of this distribution is equal for ISIs and instantaneous rate perspective.

### 3.4. Distribution Involving a Refractory Period

The refractory period is a state of the neuron, occurring right after a spike, where it is impossible for another spike to be emitted. A shifted exponential distribution function is used as an ISI descriptor for neurons with refractory period τ (Reeke and Coop, 2004). The probability density function of the shifted exponential function with parameter *a* > 0 and refractory period τ ≥ 0 is

(47) $$\begin{array}{l} f_{T}\left(t\right)=\{\begin{array}{ll} 0, & t\leq \tau \\ ae^{-a\left(t-\tau \right)}, & t>\tau \end{array} \end{array}$$

with

(48) $$\begin{array}{l} \lambda =\frac{a}{1+a\tau },\;C_{V}\left(T\right)=\frac{1}{1+a\tau }. \end{array}$$

We observe that *C*_*V*_(*T*) < 1 for *a* > 0 and τ > 0. The differential entropy for the shifted exponential distribution is evaluated as

(49) $$\begin{array}{l} h\left(f_{T}\right)=\log\left(\frac{e}{a}\right), \end{array}$$

and substituting these values into Equation (11), we arrive at

(50) $$\begin{array}{l} C_{h}\left(T\right)=\frac{1}{1+a\tau }. \end{array}$$

The pdf of the instantaneous rate is,

(51) $$\begin{array}{l} f_{R}\left(r\right)=\{\begin{array}{ll} 0, & r\geq 1/\tau \\ \frac{a^{2}}{r^{3}\left(1+a\tau \right)}e^{-a\left(\frac{1}{r}-\tau \right)}, & r<1/\tau \end{array} \end{array}$$

with

(52) $$\begin{array}{l} C_{V}\left(R\right)=\sqrt{\left(1+a\tau \right)e^{a\tau }\Gamma \left(0,a\tau \right)-1} \end{array}$$

where $\Gamma \left(s,x\right)=\int_{x}^{\infty }t^{s-1}e^{-t}\text{d}t$ is the upper incomplete gamma function (Abramowitz and Stegun, 1972).

Evaluation of the differential entropy from Equation (9) yields,

(53) $$\begin{array}{l} h\left(f_{R}\right)=-\log\left(\frac{a^{2}}{1+a\tau }\right)-\frac{3\left(1+e^{a\tau }\Gamma \left(0,a\tau \right)+\left(1+a\tau \right)\log\tau \right)}{1+a\tau }\\ \;+\frac{2+a\tau }{1+a\tau } \end{array}$$

Expression for the dispersion coefficient is derived through Equation (11),

(54) $$\begin{array}{l} C_{h}\left(R\right)=\frac{1+a\tau }{a}\exp\{-\log\left(\frac{a^{2}}{1+a\tau }\right)\\ \;-\frac{3\left(1+e^{a\tau }\Gamma \left(0,a\tau \right)+\left(1+a\tau \right)\log\tau \right)}{1+a\tau }+\frac{2+a\tau }{1+a\tau }-1\}. \end{array}$$

For the shifted exponential distribution, we observe that from Equation (48),

(55) $$\begin{array}{l} a=\frac{\lambda }{1-\lambda \tau }, \end{array}$$

which leads to,

(56) $$\begin{array}{l} C_{V}\left(T\right)=1-\lambda \tau , \end{array}$$

i.e., *C*_*V*_(*T*) depends on λ and τ. Hence, in order to analyze the relationship between *C*_*V*_(*T*) and *C*_*V*_(*R*) we have to vary the values of λ and τ (Figure 2).

Substituting the values from Equations (55) and (56) into Equation (54), we get

(57) $$\begin{array}{l} C_{V}\left(R\right)\\ =\sqrt{\left(1+\frac{1-C_{V}\left(T\right)}{C_{V}\left(T\right)}\right)\exp\left(\frac{1-C_{V}\left(T\right)}{C_{V}\left(T\right)}\right)\Gamma \left(0,\frac{1-C_{V}\left(T\right)}{C_{V}\left(T\right)}\right)-1} \end{array}$$

The dispersion coefficient of randomness *C*_*h*_(*R*) increases until it attains its maximum value 0.8137 for *C*_*V*_(*T*) = 0.85, whereas *C*_*h*_(*T*) keeps increasing monotonously. This behavior can be better understood by looking at the behavior of *C*_*h*_(*R*) while *C*_*V*_(*T*) increases in **Figure 6C**. It is observed that *C*_*h*_(*R*) attains its maximum value and then declines as *C*_*V*_(*T*) → 1, whereas *C*_*h*_(*T*) monotonously increases.

### 3.5. Mixture of Two Exponential Distributions With Refractory Period and Application to Experimental Data

Throughout the years, empirical studies have produced evidence of bimodal or multimodal trends in ISI data (Rodieck et al., 1962; Nakahama et al., 1968; Obeso et al., 2000; Dorval et al., 2008). The underlying assumption is that a neuron might be in one of the several “states,” with each state being characterized by a different ISI distribution (Tuckwell, 1988). A mixture of two distributions is commonly used to modal such data, for example, Bhumbra and Dyball (2004) used a mixture of two lognormal distributions as an ISI descriptor of supraoptic nucleus neurons, and recently gamma-exponential mixture distribution has been used to characterize the ISI distribution in the auditory system (Heil et al., 2007; Neubauer et al., 2009).

In this section, we consider the mixture of two exponential distributions (Tuckwell, 1989), which is often used to describe the bursting activity in neurons, where a sequence of short ISIs is dispersed among comparatively longer ISIs. In 1965, Smith and Smith (1965) used the mixture of two exponential distributions to explain the bursting activity in the isolated cortical neurons of an unanesthetized cat. Thomas (1966) used mixed exponential distributions to describe the ISIs in their study of the clustered firing of cortical neurons. Trapani and Nicolson (2011) found that in the lateral line organs of a zebrafish, when the depolarizing currents were blocked, the ISI data of afferent neurons was best described by a mixture of exponential distributions.

The pdf of the mixed exponential distribution with refractory period τ ≥ 0 and mixture components with parameter *a* > 0, *b* > 0, *a* ≠ *b* is given by

(58) $$\begin{array}{l} f_{T}\left(t\right)=\{\begin{array}{ll} 0, & t\leq \tau \\ pae^{-a\left(t-\tau \right)}+\left(1-p\right)be^{-b\left(t-\tau \right)}, & t>\tau \end{array} \end{array}$$

where *p* ∈ (0, 1). In this case,

(59) $$\begin{array}{l} \lambda =\frac{ab}{pb\left(1+a\tau \right)+\left(1-p\right)a\left(1+b\tau \right)}. \end{array}$$

**Figure 4:**
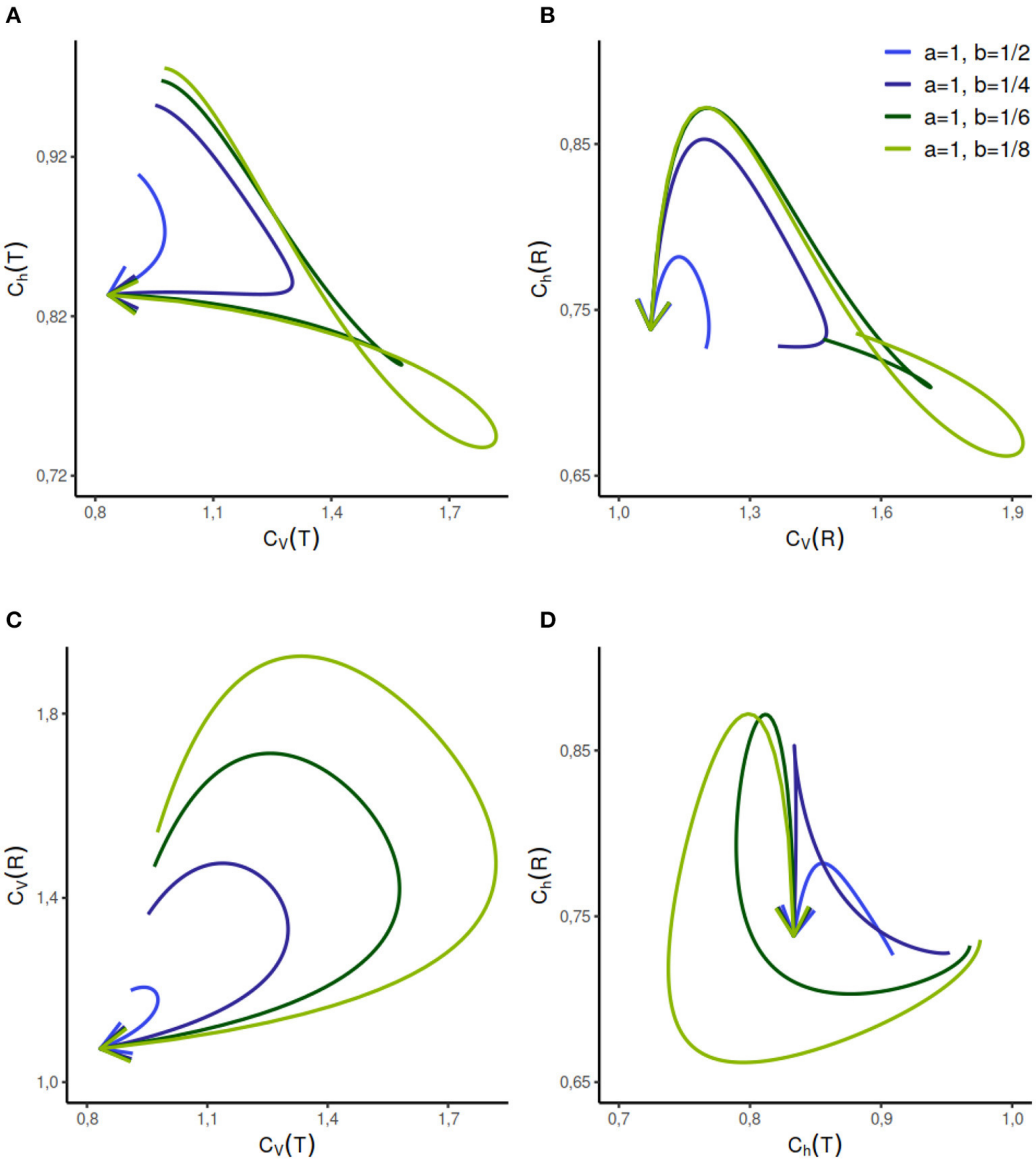
Dispersion measures of the mixed exponential distribution with refractory period randomly set to τ = 0.2 and variable weight of components as *p* ∈ [0, 1] in the direction of the arrows. The rate parameter of one component is fixed at *a* = 1 whereas the rate parameter *b* of the second component varies. When *p* = 0 or *p* = 1, the distribution is shifted exponential with refractory period τ. The dispersion measure of randomness *C*_*h*_(*T*) describes *C*_*V*_(*T*) uniquely when the *a* = 1, *b* = 1/2, whereas the reverse is not true **(A)**. This relationship gets complicated with the increasing separation between the rate parameters of the components. As seen in **(B–D)**, the relationship between the other dispersion measures is relatively complicated and non-unique for the given set of parameters.

The analytical expressions for the *C*_*V*_s and *C*_*h*_s are difficult to obtain, however, they can be calculated numerically for a given set of parameters (Figure 4). The parameter range for this distribution can be vast, we analyze the dispersion coefficients for a few different sets of component rate parameters *a* and *b*, given a value of τ and *p* ∈ [0, 1]. We study the change in randomness and variability as probability variable *p* increases in the direction of the arrows. The behavior of this model is relatively complicated but for selected cases, such as when *a* = 1, *b* = 1/2, or *a* = 1, *b* = 1/4, different firing regimes are uniquely described by *C*_*h*_(*T*) but not by *C*_*V*_(*T*).

**Figure 5:**
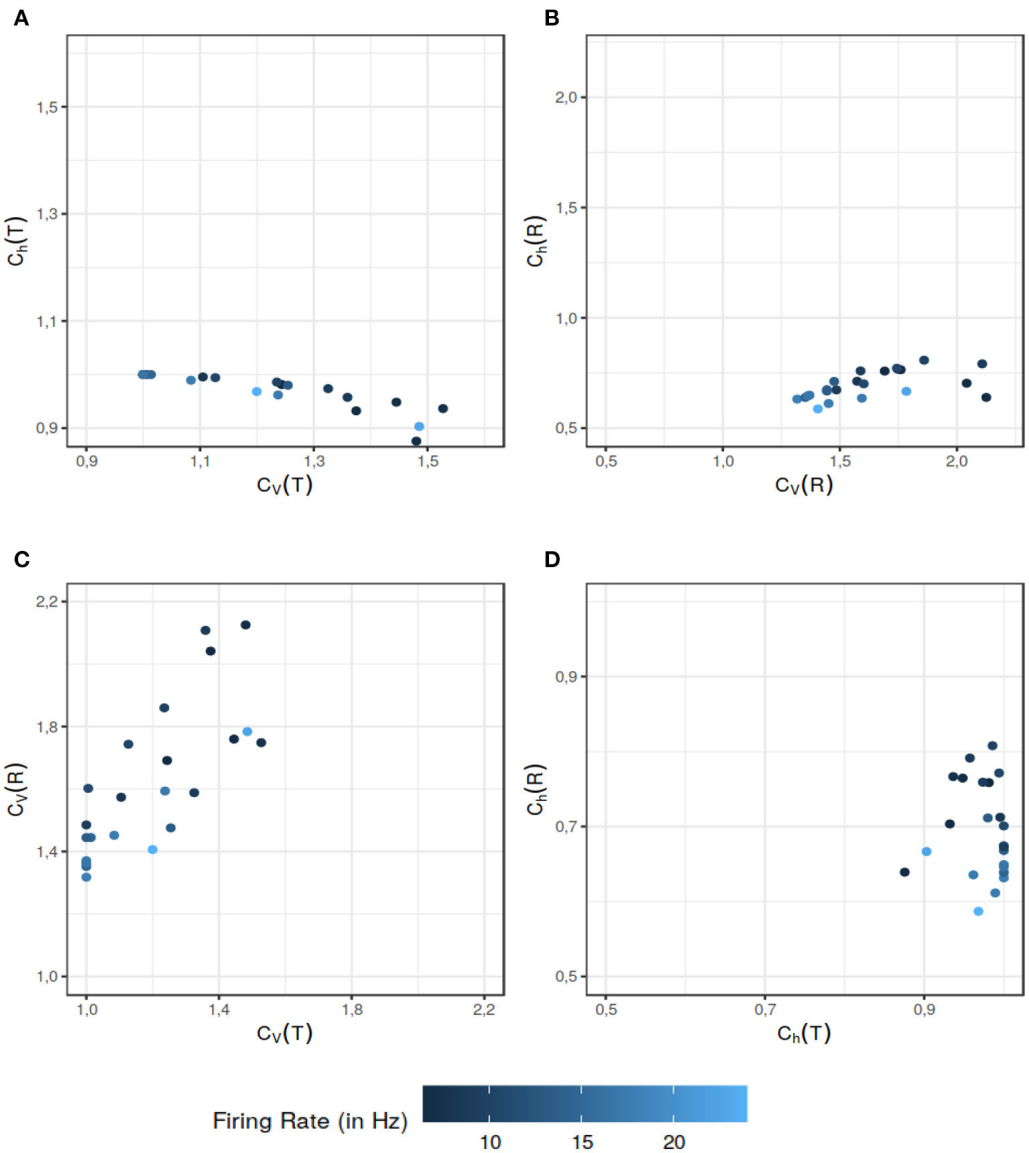
Comparison between the dispersion coefficients of variability and randomness of the experimental data for the data sets used in Song et al. (2018). The ISI data has two components: the refractory period and the hair-cell synaptic release time (the time to excitation input). Latter is modeled by a mixture distribution of two exponentials. The color gradient indicates the mean firing rate of the particular data set. The comparison of various dispersion quantities reveal various aspect to differentiate the data sets. As seen in **(A)**, *C*_*h*_(*T*) classifies the data sets in a similar category from the randomness perspective even if their ISI variability are on a wider scale. The dispersion measure of variability *C*_*V*_(*R*) also does a better job of differentiating among the data sets in **(B)** when their randomness dispersion measure would put them in a similar category. In **(C)**, *C*_*V*_(*T*) and *C*_*V*_(*R*) reveal separate aspects of the data sets. Overall *C*_*V*_(*R*) helps differentiate among the data sets with similar *C*_*V*_(*T*) values. Finally in **(D)** we can see that *C*_*h*_(*R*) further differentiates the data sets with equal *C*_*h*_(*T*). The data sets which might have similar randomness on the ISI scale, can be differentiated on the basis of their instantaneous rate randomness. All of these figures support our assertion that dispersion measures of instantaneous rate provide additional information that can be helpful in distinguishing the data sets.

Song et al. (2018) model the spike generation in the spontaneously active afferent neurons of the Zebrafish lateral line, as a renewal process. The authors propose that a spike is generated if the neuron is recovered from the refractory period *and* a synaptic release (excitatory input) from hair cells has arrived and thus ISI *T* = τ + *T*_*E*_ where τ is the absolute refractory period and *T*_*E*_ is the time to excitation (we omit the small relative refractory period used in the original paper, for computational simplicity). It is demonstrated in the paper that the mixture of exponential distributions used to model the hair cell synaptic release time *T*_*E*_, yields the best fit for the ISI data. The pdf of the ISI, in this case is given by Equation (58). We calculated *C*_*h*_ and *C*_*V*_ for ISIs and the instantaneous rate of each data set fitted with a combination of absolute refractory period and mixture of exponential distributions. For the given data sets, *C*_*V*_(*T*) and *C*_*V*_(*R*) offer more information than their randomness counterparts *C*_*h*_(*T*) (Figure 5A) and *C*_*h*_(*R*) (Figure 5B) respectively. When it comes to the dispersion measures of variability, as seen in Figure 5C, in some situations *C*_*V*_(*R*) offers additional information to distinguish further nuances in the data. The data sets which might seem of similar ISI variability, differ when it comes to the variability of their instantaneous rate. Similar results follow for the randomness (Figure 5D). This example supports our assertion that the dispersion measures based on the instantaneous rate can provide additional information to help differentiate among the data sets.

## 4. Discussion

We studied the spiking activity described by the renewal processes from two perspectives, the temporal point of view (in terms of ISIs) and the frequency point of view (in terms of instantaneous rate). We found that for a given spike train the temporal characteristics and the instantaneous rate characteristics, can either follow the same trend or opposite trends. This is due to the fact that the instantaneous rate distribution is obtained from length-biased sampling of ISIs (Equation 3). Spike trains described by non-renewal processes have been studied widely (Eden and Kass, 2016) but are beyond the scope of this paper. For the special case of serially correlated ISIs, the results of our analysis apply to marginal distributions and therefore remain unchanged (Kostal and Lansky, 2006).

**Figure 6:**
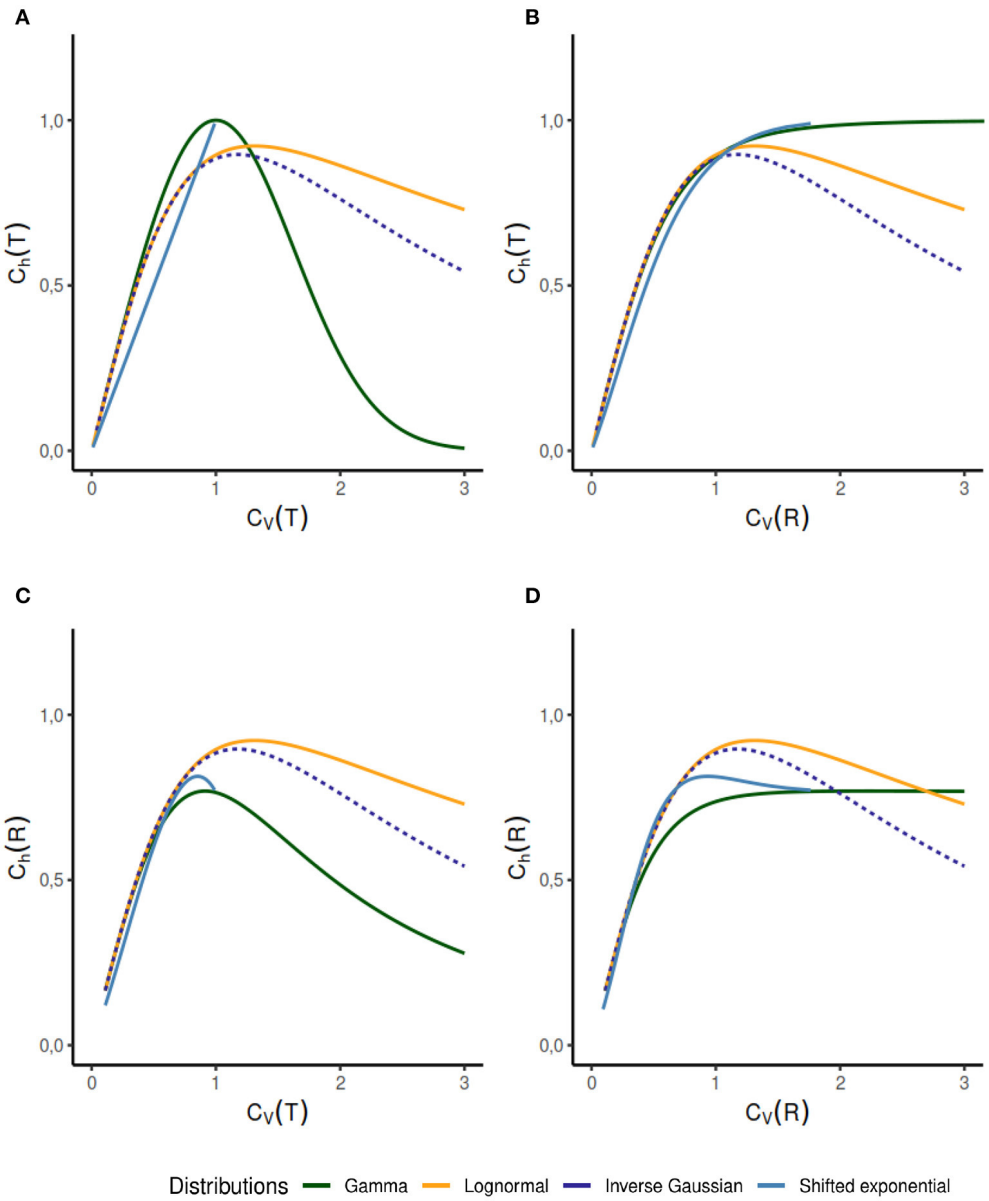
Dispersion measures of the gamma, lognormal, inverse Gaussian, and shifted exponential distributions. Each randomness dispersion measure *C*_*h*_(*R*) and *C*_*h*_(*T*) is categorized by their dependence on ISI variability *C*_*V*_(*T*) and on instantaneous rate variability *C*_*h*_(*T*). Firing rate is fixed at λ = 1 for the scale family distributions. For the gamma distribution, *C*_*h*_(*T*) declines as a function of *C*_*V*_(*T*) **(A)** but increases as a function of *C*_*V*_(*R*) **(B)**. For lognormal and inverse Gaussian distribution *C*_*h*_(*T*) = *C*_*h*_(*R*), regardless of the perspective change **(A–D)**. The shifted exponential distribution has a monotonously increasing *C*_*h*_(*T*), when it is a function of *C*_*V*_(*T*) or *C*_*V*_(*R*) **(A,B)**; whereas *C*_*h*_(*R*) attains its maximum for *C*_*V*_(*T*) = 0.85 and *C*_*V*_(*R*) = 0.9282 respectively, and then declines **(C,D)**.

1. We observe several different relationships between the variability from temporal and instantaneous rate perspectives (Figure 2). For gamma distribution, the variability of instantaneous rate is higher than the variability in ISIs whereas for lognormal and inverse Gaussian distribution it stays the same, *C*_*V*_(*T*) = *C*_*V*_(*R*). On the other hand, for shifted exponential distribution *C*_*V*_(*R*) < *C*_*V*_(*T*) until *C*_*V*_(*T*) = 0.7715 but after that *C*_*V*_(*R*) increases rapidly compared to *C*_*V*_(*T*).
2. In the case of gamma distribution, both the randomness measures *C*_*h*_(*T*) and *C*_*h*_(*R*) decline eventually as a function of *C*_*V*_(*T*) but they become constant as a function of *C*_*V*_(*R*) (Figures 6A–D). The randomness for gamma distribution differs in each particular case, and for small values of *C*_*V*_(*T*), *C*_*h*_(*R*) < *C*_*h*_(*T*). Lognormal and inverse Gaussian distributions attain their maximum for *C*_*V*_(*T*) values close to 1 and then their randomness keeps declining (Figures 6A,C). For these two distributions, *C*_*h*_(*T*) = *C*_*h*_(*R*) whether it is mapped against *C*_*V*_(*T*) or *C*_*V*_(*R*). For shifted exponential distribution, *C*_*h*_(*T*) increases as a function of *C*_*V*_(*T*) (Figure 6A) whereas *C*_*h*_(*R*) attains its maximum for *C*_*V*_(*T*) = 0.85 and then declines fast as *C*_*V*_ → 1 (Figure 6C).
3. We studied the case of mixed exponential distribution with a refractory period. Although the theoretical analysis is complicated, we use the experimental data obtained from Song et al. (2018) to inspect the temporal and instantaneous rate perspectives. The dispersion measures for instantaneous rate provide a novel outlook on the data, different from the one provided by the dispersion measures for the ISIs (Figure 6). In cases like these, the instantaneous rate may be helpful in distinguishing further nuances in the data.

## Data Availability Statement

Publicly available datasets were analyzed in this study. This data can be found at: https://static-content.springer.com/esm/art%3A10.1038%2Fs41598-018-33064-z/MediaObjects/41598_2018_33064_MOESM1_ESM.pdf.

## Author Contributions

LK proposed the concept of the article. RT and LK wrote the manuscript. RT performed the calculations, numerical simulations, and analysis. All authors contributed to the article and approved the submitted version.

## Conflict of Interest

The authors declare that the research was conducted in the absence of any commercial or financial relationships that could be construed as a potential conflict of interest.
