# Supplementary material for: Variability and Randomness of the Instantaneous Firing Rate
Source: Front Comput Neurosci. 2021 Jun 7;15:620410. doi: 10.3389/fncom.2021.620410 (PMC8215133; doi:10.3389/fncom.2021.620410)
Supplement: Supplementary file 1 [file Data_Sheet_1.PDF]

Supplementary Material

1 GAMMA DISTRIBUTION

The pdf of Gamma distribution is given by Equation 13. Our aim is to derive the expression for differential entropy of this distribution. From Equation 9 we know that,

$$
\begin{aligned}
h(f_T) &= -\int_0^\infty f_T(t)\log(f_T(t))\mathrm{d}t \\
&= -\int_0^\infty f_T(t)\log\left(\frac{b^a t^{a-1}e^{-bt}}{\Gamma(a)}\right)\mathrm{d}t \\
&= -\log\left(\frac{b^a}{\Gamma(a)}\right)\int_0^\infty f_T(t)\mathrm{d}t - (a-1)\int_0^\infty \log(t) f_T(t)\mathrm{d}(t) + b\int_0^\infty t f_T(t)\mathrm{d}t \\
&= -\log\left(\frac{b^a}{\Gamma(a)}\right) - \frac{(a-1)b^a}{\Gamma(a)}\int_0^\infty \log(t)t^{a-1}e^{-bt}\mathrm{d}t + \frac{b^{a+1}}{\Gamma(a)}\int_0^\infty t^a e^{-bt}\mathrm{d}t,
\end{aligned}
$$

The integrals in the second and third term can be solved by linear substitution and we arrive at the simplified for of $h(f_T)$,

$$
\begin{aligned}
h(f_T) &= -\log\left(\frac{b^a}{\Gamma(a)}\right) - \frac{(a-1)}{\Gamma(a)}[\Gamma'(a) - \log(b)\Gamma(a)] + \frac{1}{\Gamma(a)}\Gamma(a+1) \\
&= \log\Gamma(a) - \log(b) - (a-1)\psi(a) + a \\
&= \log\left(\frac{\Gamma(a)}{b}e^{a+(1-a)\psi(a)}\right)
\end{aligned}
$$

where $\Gamma(x) = \int_0^\infty z^{x-1}e^{-z}\mathrm{d}z$ is the gamma function and $\psi(x) = \Gamma'(x)/\Gamma(x)$ is the digamma function.

The entropy of the instantaneous rate distribution, with pdf given in Equation 17, is calculated in a similar manner,

$$
\begin{aligned}
h(f_R) &= -\int_0^\infty f_R(r)\log f_R(r)\mathrm{d}r \\
&= -\int_0^\infty f_R(r)\log\left(\frac{b^{a+1}}{\Gamma(a+1)}r^{-a-2}e^{-b/r}\right)\mathrm{d}r \\
&= -\log\left(\frac{b^{a+1}}{\Gamma(a+1)}\right)\int_0^\infty f_R(r)\mathrm{d}r + (a+2)\int_0^\infty \log r f_R(r)\mathrm{d}(r) + b\int_0^\infty \frac{1}{r}f_R(r)\mathrm{d}r \\
&= -\log\left(\frac{b^{a+1}}{\Gamma(a+1)}\right) + \frac{(a+2)}{\Gamma(a+1)}\int_0^\infty b^{a+1}r^{-a-2}e^{-b/r}\log r\mathrm{d}r + \frac{1}{\Gamma(a+1)}\int_0^\infty b^{a+2}r^{-a-3}e^{-b/r}\mathrm{d}r \\
&= -\log\left(\frac{b^{a+1}}{\Gamma(a+1)}\right) + \frac{(a+2)}{\Gamma(a+1)}\int_0^\infty \left(\frac{b}{r}\right)^{a+1}\frac{1}{r}e^{-b/r}\log r\mathrm{d}r + \frac{1}{\Gamma(a+1)}\int_0^\infty \left(\frac{b}{r}\right)^{a+2}\frac{1}{r}e^{-b/r}\mathrm{d}r,
\end{aligned}
$$

The integrals in the second and third term are easily solved by using a substitution for b/r,

$$
\begin{aligned}
&= -\log\left(\frac{b^{a+1}}{\Gamma(a+1)}\right) + \frac{(a+2)}{\Gamma(a+1)}[\Gamma(a+1)\log b - \Gamma'(a+1)] + \frac{1}{\Gamma(a+1)}\Gamma(a+2) \\
&= -\log\left(\frac{b^{a+1}}{\Gamma(a+1)}\right) + (a+2)\log b - (a+2)\psi(a+1) + (a+1) \\
&= \log(\Gamma(a+1)be^{(a+1)-(a+2)\psi(a+1)}).
\end{aligned}
$$

To derive the coefficient of variation $C_V(R)$ from Equation 8, we first need to find an expression for $\mathbb{E}(1/T)$,

$$
\begin{aligned}
\mathbb{E}\left(\frac{1}{T}\right) &= \int_0^\infty \frac{1}{t} f_T(t)\mathrm{d}t \\
&= \int_0^\infty \frac{1}{t} \frac{b^a t^{a-1} e^{-bt}}{\Gamma(a)}\mathrm{d}t \\
&= \frac{b^a}{\Gamma(a)} \int_0^\infty t^{a-2} e^{-bt}\mathrm{d}t \\
&= \frac{b^a}{\Gamma(a)} b^{1-a}\Gamma(a-1) \\
&= \frac{b}{a-1}.
\end{aligned}
$$

Hence,

$$
\begin{aligned}
C_V(R) &= \sqrt{\frac{\mathbb{E}(1/T)}{\lambda} - 1} \\
&= \sqrt{\frac{b}{a-1}\frac{a}{b} - 1} \\
C_V(R) &= \sqrt{\frac{1}{a-1}}.
\end{aligned}
$$

2 LOGNORMAL DISTRIBUTION

Entropy of the lognormal distribution with pdf given by Equation 26, is

$$
\begin{aligned}
h(f_T) &= -\int_0^\infty f_T(t)\log(f_T(t))\mathrm{d}t \\
&= -\int_0^\infty f_T(t)\log\left(\frac{1}{\sigma t\sqrt{2\pi}}\exp\left\{-\frac{(\log t-\log m)^2}{2\sigma^2}\right\}\right)\mathrm{d}t\Bigg) \\
&= -\int_0^\infty f_T(T)\Bigg(-\log(\sigma\sqrt{(2\pi)})-\log t-\frac{(\log t-\log m)^2}{2\sigma^2}\Bigg)\mathrm{d}t \\
&= \log(\sqrt{2\pi\sigma^2})\int_0^\infty f_T(t)\mathrm{d}t+\int_0^\infty \log t f_T(t)\mathrm{d}t+\frac{1}{2\sigma^2}\int_0^\infty(\log t-\log m)^2 f_T(t)\mathrm{d}t \\
&= \log(\sqrt{2\pi\sigma^2})+\frac{1}{\sqrt{2\pi\sigma^2}}\int_0^\infty \frac{\log t}{t}\exp\left\{-\frac{(\log t-\log m)^2}{2\sigma^2}\right\}\mathrm{d}t \\
&\quad+\frac{1}{\sqrt{2\pi\sigma^2}}\int_0^\infty \frac{(\log t-\log m)^2}{2\sigma^2 t}\exp\left\{-\frac{(\log t-\log m)^2}{2\sigma^2}\right\}\mathrm{d}t
\end{aligned}
$$

To simplify it further, we substitute $(\log t - \log m) = y$ into the integrals in the second and third term,

$$
= \log(\sqrt{2\pi\sigma^2})+\frac{1}{\sqrt{2\pi\sigma^2}}\int_{-\infty}^\infty (y+\log m)e^{-y^2/2\sigma^2}\mathrm{d}y+\frac{1}{\sqrt{2\pi\sigma^2}}\int_{-\infty}^\infty \frac{y^2}{2\sigma^2}e^{-y^2/2\sigma^2}\mathrm{d}y
$$

Second term can be solved easily and to solve the third term integral, we again use the substitution method. Substituting $y/\sqrt{2}\sigma = x$ in the integral, we arrive at

$$
= \log(\sqrt{2\pi\sigma^2})+\log m+\frac{1}{\sqrt{\pi}}\int_{-\infty}^\infty x^2 e^{-x^2}\mathrm{d}x. \tag{S1}
$$

To find the value of the third term integral, let's consider

$$
I_s = \int_{-\infty}^\infty e^{-sm^2}\mathrm{d}m
$$

then,

$$
\begin{aligned}
-\frac{d}{ds}I_s|_{s=1} &= -\int_{-\infty}^\infty -sm^2 e^{-sm^2}\mathrm{d}m\ |_{s=1} \\
&= \int_{-\infty}^\infty m^2 e^{-m^2}\mathrm{d}m.
\end{aligned}
$$

The last term in the equation above corresponds to the integral in the third term of Equation S1. Now we find the value of the integral I_s,

$$
\begin{aligned}
I_s^2 &= \int_{-\infty}^{\infty} e^{-sm^2} \mathrm{d}m \int_{-\infty}^{\infty} e^{-sn^2} \mathrm{d}n \\
&= \int_{-\infty}^{\infty} \int_{-\infty}^{\infty} e^{-s(m^2+n^2)} \mathrm{d}m\mathrm{d}n,
\end{aligned}
$$

let $m = r\mathrm{Sin}\theta$ and $n = r\mathrm{Cos}\theta$,

$$
\begin{aligned}
I_s^2 &= \int_0^{2\pi} \mathrm{d}\theta \int_0^{\infty} e^{-sr^2} r\mathrm{d}r \\
&= 2\pi \int_0^{\infty} re^{-sr^2} \mathrm{d}r,
\end{aligned}
$$

this integral is easily solved by using linear substitution for r^2,

$$
\begin{aligned}
I_s^2 &= 2\pi \frac{1}{2s} \\
&= \frac{\pi}{s}
\end{aligned}
$$

Hence,

$$
I_s = \sqrt{\frac{\pi}{s}}.
$$

The integral in the third term of the Equation S1 is,

$$
\begin{aligned}
\int_{-\infty}^{\infty} x^2 e^{-x^2} \mathrm{d}x &= -\frac{d}{ds} I_s \mid_{s=1} \\
&= -\frac{d}{ds} \sqrt{\frac{\pi}{s}} \Big|_{s=1} \\
&= \frac{\sqrt{\pi}}{2}.
\end{aligned}
$$

Substituting this value back in Equation S1 gives us the expression for the ISI entropy,

$$
\begin{aligned}
h(f_T) &= \log(\sqrt{2\pi\sigma^2}) + \log m + \frac{1}{2} \\
&= \frac{1}{2} \log(2\pi e \sigma^2 m^2).
\end{aligned}
$$

The pdf of the instantaneous rate distribution is given in Equation 30 and the expression for the entropy is,

$$\begin{aligned}
h(f_R) &= -\int_0^\infty f_R(r)\log f_R(r)\mathrm{d}r \\
&= -\int_0^\infty f_R(r)\log\left(\frac{1}{me^{\sigma^2/2}}\frac{1}{r^2\sigma\sqrt{2\pi}}\exp\big\{-\frac{(\ln r+\ln m)^2}{2\sigma^2}\big\}\right)\mathrm{d}r \\
&= \log(\sqrt{2\pi}\sigma m e^{\sigma^2/2}) + 2\int_0^\infty \log r f_R(r)\mathrm{d}r + \frac{1}{2\sigma^2}\int_0^\infty(\log r-\log m)^2 f_R(r)\mathrm{d}r \\
&= \log(\sqrt{2\pi}\sigma m e^{\sigma^2/2}) + \frac{2}{\sigma m e^{\sigma^2/2}\sqrt{2\pi}}\int_0^\infty \frac{\log r}{r^2}\exp\left\{-\frac{(\log r+\log m)^2}{2\sigma^2}\right\}\mathrm{d}r \\
&\quad + \frac{1}{\sigma m e^{\sigma^2/2}\sqrt{2\pi}}\int_0^\infty \frac{(\log r+\log m)^2}{2\sigma^2 r^2}\exp\left\{-\frac{(\log r+\log m)^2}{2\sigma^2}\right\}\mathrm{d}r.
\end{aligned}$$

Substitute $\log r + \log m = y$ to simplify the integrals,

$$\begin{aligned}
&= \log(\sqrt{2\pi}\sigma m e^{\sigma^2/2}) + \frac{2}{\sqrt{2\pi}\sigma e^{\sigma^2/2}}\int_{-\infty}^\infty (y-\log m)e^{-y}e^{-y^2/2\sigma^2}\mathrm{d}y \\
&\quad + \frac{1}{\sqrt{2\pi}\sigma e^{\sigma^2/2}}\int_{-\infty}^\infty \frac{y^2}{2\sigma^2}e^{-y}e^{-y^2/2\sigma^2}\mathrm{d}y \\
&= \log(\sqrt{2\pi}\sigma m e^{\sigma^2/2}) + \frac{2}{\sigma m e^{\sigma^2/2}\sqrt{2\pi}}\int_{-\infty}^\infty ye^{-y}e^{-y^2/2\sigma^2}\mathrm{d}y \\
&\quad - \frac{2\log m}{\sigma\sqrt{2\pi}e^{\sigma^2/2}}\int_\infty^\infty e^{-y}e^{-y^2/2\sigma^2}\mathrm{d}y + \frac{1}{\sigma\sqrt{2\pi}e^{\sigma^2/2}}\int_\infty^\infty \frac{y^2}{2\sigma^2}e^{-y}e^{-y^2/2\sigma^2}\mathrm{d}y \\
&= \log(\sqrt{2\pi}\sigma m e^{\sigma^2/2}) + \frac{2}{\sigma m e^{\sigma^2/2}\sqrt{2\pi}}I_1 - \frac{2\log m}{\sigma\sqrt{2\pi}e^{\sigma^2/2}}I_2 + \frac{1}{\sigma\sqrt{2\pi}e^{\sigma^2/2}}I_3. \qquad \text{(S2)}
\end{aligned}$$

We will individually solve for the integrals I_1, I_2 and I_3. Note that,

$$e^{-y}e^{-y^2/2\sigma^2} = e^{-(\frac{y}{\sqrt{2}\sigma}+\frac{\sigma}{\sqrt{2}})^2}e^{\sigma^2/2}. \qquad \text{(S3)}$$

Using the relationship S3 in I_1,

$$\begin{aligned}
I_1 &= \int_{-\infty}^\infty ye^{-y}e^{-y^2/2\sigma^2}\mathrm{d}y \\
&= e^{\sigma^2/2}\int_{-\infty}^\infty ye^{-(\frac{y}{\sqrt{2}\sigma}+\frac{\sigma}{\sqrt{2}})^2}\mathrm{d}y.
\end{aligned}$$

This can be simplified further by the substituting $\frac{y}{\sqrt{2}\sigma}+\frac{\sigma}{\sqrt{2}} = n$ in the integral,

$$\begin{aligned}
&= \sqrt{2}\sigma\int_{-\infty}^\infty (n\sqrt{2}\sigma-\sigma^2)e^{-n^2}\mathrm{d}n \\
&= 2\sigma^2\int_{-\infty}^\infty ne^{-n^2}\mathrm{d}n - \sqrt{2}\sigma^3\int_{-\infty}^\infty e^{-n^2}\mathrm{d}n.
\end{aligned}$$

The first term has an odd integrand, hence equates to zero. The second term is the Gaussian integral $\int_{-\infty}^{\infty} e^{-n^2} \mathrm{d}n = \sqrt{\pi}$ (Weisstein, 2020). Hence,

$$I_1 = -\sqrt{2\pi}\sigma^3. \tag{S4}$$

We use Equation S3 to simplify and solve the integral I_2 in the following way,

$$\begin{aligned} I_2 &= \int_{-\infty}^{\infty} e^{-y} e^{-y^2/2\sigma^2} \mathrm{d}y \\ &= e^{\sigma^2/2} \int_{-\infty}^{\infty} e^{(\frac{y}{\sqrt{2}\sigma} + \frac{\sigma}{\sqrt{2}})^2} \mathrm{d}y \\ &= e^{\sigma^2/2} \int_{-\infty}^{\infty} e^{\frac{1}{2\sigma^2}(y+\sigma^2)^2} \mathrm{d}y \\ &= \sigma\sqrt{2\pi} e^{\sigma^2/2}. \end{aligned} \tag{S5}$$

Integral I_3 in the last term of Equation S2 is solved through a slightly different approach,

$$\begin{aligned} I_3 &= \int_{-\infty}^{\infty} \frac{y^2}{2\sigma^2} e^{-(y+\frac{y^2}{2\sigma^2})} \mathrm{d}y \\ &= \int_{-\infty}^{\infty} \frac{y^2}{2\sigma^2} e^{-\frac{1}{2\sigma^2}(t+\sigma^2)^2 + \frac{1}{2}\sigma^2} \mathrm{d}y, \end{aligned}$$

let $z = y + \sigma^2$,

$$\begin{aligned} &= \frac{1}{2\sigma^2} e^{\sigma^2/2} \int_{-\infty}^{\infty} (z-\sigma^2)^2 e^{-\frac{z^2}{2\sigma^2}} \mathrm{d}z \\ &= \frac{1}{2\sigma^2} e^{\sigma^2/2} \int_{-\infty}^{\infty} (z^2+\sigma^4) e^{-\frac{z^2}{2\sigma^2}} \mathrm{d}z. \end{aligned}$$

Let $a = 1/2\sigma^2$ and use $J(a) = \int_{-\infty}^{\infty} e^{-az^2} \mathrm{d}z = \sqrt{\frac{\pi}{a}}, \quad \int_{-\infty}^{\infty} z^2 e^{-az^2} \mathrm{d}z = -J'(a) = \frac{\sqrt{\pi}}{2a^{3/2}}$, to obtain

$$\begin{aligned} I_3 &= \frac{1}{2\sigma^2} e^{\sigma^2/2} (\sqrt{2\pi}\sigma^3 + \sqrt{2\pi}\sigma^5) \\ &= \sqrt{\frac{\pi}{2}} e^{\sigma^2/2} \sigma(1+\sigma^2). \end{aligned} \tag{S6}$$

Plug the values from Equation S4, S5 and S6 into Equation S2 to get

$$\begin{aligned} h(f_R) &= \log(\sqrt{2\pi}\sigma m e^{\sigma^2/2}) - 2\sigma^2 - 2\log m + \frac{1}{2}(\sigma^2+1) \\ &= \frac{1}{2} \log \frac{1}{m} 2\pi\sigma^2 e^{1-2\sigma^2}. \end{aligned}$$

To derive the expression for $C_V(R)$, we first find the expression for $\mathbb{E}(1/T)$,

$$\begin{aligned}\mathbb{E}\left(\frac{1}{T}\right) &= \int_0^\infty \frac{1}{t} f_T(t)\mathrm{d}t \\ &= \int_0^\infty \frac{1}{t}\frac{1}{\sigma\sqrt{2\pi}} \exp\left\{-\frac{(\log t - \log m)^2}{2\sigma^2}\right\}\mathrm{d}t \\ &= \frac{1}{\sigma\sqrt{2\pi}} \int_0^\infty \frac{1}{t^2} \exp\left\{-\frac{(\log t - \log m)^2}{2\sigma^2}\right\}\mathrm{d}t,\end{aligned}$$

let $(\log t - \log m) = y$,

$$\begin{aligned}&= \frac{1}{\sigma\sqrt{2\pi}} \int_{-\infty}^\infty \frac{1}{me^y} e^{-y^2/2\sigma^2}\mathrm{d}y \\ &= \frac{1}{m\sigma\sqrt{2\pi}} \int_{-\infty}^\infty e^{-y} e^{-y^2/2\sigma^2}\mathrm{d}y,\end{aligned}$$

This is the previously mentioned integral I_2, which is solved in Equation S5, substituting the value of I_2, we arrive at

$$\begin{aligned}&= \frac{1}{m\sigma\sqrt{2\pi}}\sigma\sqrt{2\pi}e^{\sigma^2/2} \\ &= \frac{e^{\sigma^2/2}}{m}.\end{aligned}$$

Now, we can derive $C_V(R)$ from Equation 8,

$$\begin{aligned}C_V(R) &= \sqrt{\frac{\mathbb{E}(1/T)}{\lambda} - 1} \\ &= \sqrt{\frac{e^{\sigma^2/2}}{m}\frac{me^{\sigma^2/2}}{1} - 1} \\ &= \sqrt{e^{\sigma^2} - 1}.\end{aligned}$$

3 INVERSE GAUSSIAN DISTRIBUTION

Entropy of the ISI distribution with pdf given by Equation 37,

$$\begin{aligned} h(f_T) &= -\int_0^\infty f_T(t)\log(f_T(t))\mathrm{d}t \\ &= \frac{1}{2}\log\frac{2\pi b}{a}\int_0^\infty f_T(t)\mathrm{d}t + \frac{3}{2}\int_0^\infty \log t f_T(t)\mathrm{d}t + \frac{1}{2b}\int_0^\infty \frac{(t-a)^2}{at} f_T(t)\mathrm{d}t \\ &= \frac{1}{2}\log\frac{2\pi b}{a} + \sqrt{\frac{a}{2\pi b}}\, e^{1/b}\left[\frac{3}{2}\int_0^\infty \log t\, t^{-\frac{3}{2}}\, e^{-\frac{1}{2b}\left(\frac{t}{a}+\frac{a}{t}\right)}\mathrm{d}t \right. \\ &\quad \left. + \frac{1}{2b}\int_0^\infty \left(\frac{t}{a} - 2 + \frac{a}{t}\right) t^{-\frac{3}{2}}\, e^{-\frac{1}{2b}\left(\frac{t}{a}+\frac{a}{t}\right)}\mathrm{d}t\right]. \end{aligned}$$

Note that we can write all integrals in $h(f_T)$ in terms of the master integral $J(a,b,n) := \frac{1}{2}\int_0^\infty t^{n-1}e^{-\frac{1}{2b}\left(\frac{t}{a}+\frac{a}{t}\right)}\mathrm{d}t$. Using this definition,

$$\begin{aligned} h(f_T) &= \frac{1}{2}\log\frac{2\pi b}{a} + \sqrt{\frac{a}{2\pi b}}\, e^{\frac{1}{b}}\left[3\frac{\partial}{\partial n}J(a,b,n)\bigg|_{n=-\frac{1}{2}} \right. \\ &\quad \left. + \frac{1}{b}\left(\frac{1}{a}J\left(a,b,\tfrac{1}{2}\right) - 2J\left(a,b,-\tfrac{1}{2}\right) + aJ\left(a,b,-\tfrac{3}{2}\right)\right)\right]. \end{aligned} \tag{S7}$$

To work with the master integral, first change variables so that $t = ae^x$. Under this transformation, $t^{n-1}dt = a^n e^{nx}dx$, and $\frac{1}{2}\left(\frac{t}{a}+\frac{a}{t}\right) = \cosh x$, and the limits change to $\pm\infty$. We arrive at,

$$J(a,b,n) = \frac{a^n}{2}\int_{-\infty}^\infty e^{nx}\, e^{\frac{1}{b}\cosh t}\mathrm{d}x = a^n\int_0^\infty \cosh(nx)e^{\frac{1}{b}\cosh t}\mathrm{d}x = a^n\mathrm{K}_n\left(\tfrac{1}{b}\right),$$

where in the second equality we split the integral into two terms, the first ranging over $[-\infty, 0]$ and the second over $[0,\infty]$, and change variables from x to $-x$ in the first term. The final equality uses a standard integral representation for the Bessel K function, as in (DLMF, 2020, Eq. 10.32.9). Substituting for this form of the master integral into Equation S7, we arrive at,

$$\begin{aligned} h(f_T) &= \frac{1}{2}\log\frac{2\pi b}{a} + \frac{1}{\sqrt{2\pi b}}\, e^{1/b}\left[3\log a\,\mathrm{K}_{\frac{1}{2}}\left(\tfrac{1}{b}\right) + 3\frac{\partial}{\partial n}\mathrm{K}_n\left(\tfrac{1}{b}\right)\bigg|_{n=-\frac{1}{2}} \right. \\ &\quad \left. + \frac{1}{b}\left(\mathrm{K}_{\frac{1}{2}}\left(\tfrac{1}{b}\right) - 2\mathrm{K}_{-\frac{1}{2}}\left(\tfrac{1}{b}\right) + \mathrm{K}_{-\frac{3}{2}}\left(\tfrac{1}{b}\right)\right)\right]. \end{aligned} \tag{S8}$$

To simplify further, we require some identities for the Bessel K function. The following identities are found in (DLMF, 2020, Eq. 10.27.3, Eq. 10.39.2 and Eq. 10.38.7),

$$\mathrm{K}_{n}\left(z\right) = \mathrm{K}_{-n}\left(z\right), \tag{S9}$$

$$\mathrm{K}_{\frac{1}{2}}\left(z\right) = \sqrt{\frac{\pi}{2z}}e^{-z}, \tag{S10}$$

$$\mathrm{K}_{\frac{3}{2}}\left(z\right) = \sqrt{\frac{\pi}{2z}}\left(1+\frac{1}{z}\right)e^{-z}. \tag{S11}$$

Simplifying $h(f_T)$ using these results,

$$\begin{aligned} h(f_T) &= \frac{1}{2}\log\frac{2\pi b}{a} + \frac{3}{2}\log a + \frac{3e^{1/b}}{\sqrt{2\pi b}}K^{(1,0)}\bigg(-\frac{1}{2},\frac{1}{b}\bigg), \\ &= \frac{1}{2}\log(2\pi a^2 be) + \frac{3e^{1/b}}{\sqrt{2\pi b}}K^{(1,0)}\bigg(-\frac{1}{2},\frac{1}{b}\bigg). \end{aligned}$$

The pdf of the instantaneous rate distribution is given by Equation 42, and we note that if ISI $T \sim \mathrm{IG}(a,b)$ where $\mathrm{IG}(a,b)$ is the inverse Gaussian distribution with mean $a>0$ and scale parameter $b>0$ then $R \sim \mathrm{IG}(1/a,b)$. We can derive $h(f_R)$ through this relationship,

$$h(f_R) = \frac{1}{2}\log\left(\frac{2\pi be}{a^2}\right) + \frac{3e^{1/b}}{\sqrt{2\pi b}}K^{(1,0)}\bigg(-\frac{1}{2},\frac{1}{b}\bigg).$$

Next, we find out the expression for $\mathbb{E}(1/T)$ for the inverse Gaussian distribution,

$$\begin{aligned} \mathbb{E}\bigg(\frac{1}{T}\bigg) &= \int_0^\infty \frac{1}{t}f_T(t)\mathrm{d}t \\ &= \int_0^\infty \frac{1}{t}\sqrt{\frac{a}{2\pi bt^3}}\exp\bigg\{-\frac{1}{2b}\frac{(t-a)^2}{at}\bigg\}\mathrm{d}t, \end{aligned}$$

the integral part can be written in form of the master integral $J(a,b,n)$,

$$\begin{aligned} &= \sqrt{\frac{2a}{2\pi b}}e^{1/b}J(a,b,-3/2) \\ &= \sqrt{\frac{2a}{2\pi b}}e^{1/b}a^{-3/2}K_{-3/2}\left(\frac{1}{b}\right) \end{aligned}$$

Using Equation S9 and S11, we get

$$= \frac{1+b}{a}$$

The value of $\mathbb{E}(1/T)$ is substituted into the equation below to derive an expression for $C_V(R)$,

$$\begin{aligned} C_V(R) &= \sqrt{\frac{\mathbb{E}(1/T)}{\lambda} - 1} \\ &= \sqrt{\frac{(1+b)}{a}a - 1} \\ &= \sqrt{b}. \end{aligned}$$

4 SHIFTED EXPONENTIAL DISTRIBUTION

The pdf of the shifted exponential distribution is given in Equation 47, and the entropy of this distribution is,

$$\begin{aligned} h(f_T) &= -\int_\tau^\infty f_T(t)\log(f_T(t))\mathrm{d}t \\ &= -\int_\tau^\infty f_T(t)\log(ae^{-a(t-\tau)})\mathrm{d}t \\ &= -\log a\int_\tau^\infty f_T(t)\mathrm{d}t + a\int_\tau^\infty (t-\tau)ae^{-a(t-\tau)}\mathrm{d}t. \end{aligned}$$

Using the substitution $a(t-\tau) = x$ in the second term integral, the limit changes to $[0, \infty]$

$$\begin{aligned} &= -\log a + \int_0^\infty xe^{-x}\mathrm{d}x \\ &= \log\left(\frac{1}{a}\right) + 1 \\ &= \log\left(\frac{e}{a}\right). \end{aligned}$$

Entropy of the instantaneous rate distribution with pdf given in Equation 51,

$$\begin{aligned} h(f_R) &= -\int_0^{1/\tau} f_R(r)\log f_R(r)\mathrm{d}r \\ &= -\int_0^{1/\tau} f_R(r)\log\left(\frac{a^2}{1+a\tau}\frac{1}{r^3}e^{-a(\frac{1}{r}-\tau)}\right)\mathrm{d}r \\ &= -\log\left(\frac{a^2}{1+a\tau}\right)\int_0^{1/\tau} f_R(r)\mathrm{d}r + 3\int_0^{1/\tau}\log r f_R(r)\mathrm{d}r + a\int_0^{1/\tau}\left(\frac{1}{r}-\tau\right)f_R(r)\mathrm{d}r \\ &= -\log\left(\frac{a^2}{1+a\tau}\right) + \frac{3a^2}{1+a\tau}\int_0^{1/\tau}\frac{logr}{r^3}e^{-a(\frac{1}{r}-\tau)}\mathrm{d}r + \frac{a^3}{(1+a\tau)}\int_0^{1/\tau}\frac{1}{r^3}\left(\frac{1}{r}-\tau\right)e^{-a(\frac{1}{r}-\tau)}\mathrm{d}r \\ &= -\log\left(\frac{a^2}{1+a\tau}\right) + \frac{3a^2}{1+a\tau}I_4 + \frac{a^3}{(1+a\tau)}I_5. \end{aligned} \quad \text{(S12)}$$

Solving the integrals in the second and third term with the help of MATHEMATICA (Wolfram Research, Inc., 2020),

$$I_4 = -\frac{1 + e^{a\tau}\Gamma(0, a\tau) + (1 + a\tau)\log\tau}{a^2},$$
$$I_5 = \frac{2 + a\tau}{a^3}.$$

Substitute these values back in Equation S12,

$$h(f_R) = -\log\left(\frac{a^2}{1 + a\tau}\right) - \frac{3(1 + e^{a\tau}\Gamma(0, a\tau) + (1 + a\tau)\log\tau)}{1 + a\tau} + \frac{2 + a\tau}{1 + a\tau},$$

where $\Gamma(s, x) = \int_x^\infty t^{s-1}e^{-t}\mathrm{d}t$ is the upper incomplete gamma function. For the next step, we derive the expression for $\mathbb{E}(1/T)$,

$$\begin{aligned}
\mathbb{E}\left(\frac{1}{T}\right) &= \int_\tau^\infty \frac{1}{t} f_T(t)\mathrm{d}t \\
&= \int_\tau^\infty \frac{1}{t} ae^{-a(t-\tau)}\mathrm{d}t \\
&= ae^{a\tau}\int_\tau^\infty t^{-1}e^{-at}\mathrm{d}t \\
&= a^2e^{a\tau}\int_{tau}^\infty (at)^{-1}e^{-at}\mathrm{d}t \\
&= ae^{a\tau}\int_{a\tau}^\infty x^{-1}e^{-x}\mathrm{d}x \\
&= ae^{a\tau}\Gamma(0, a\tau).
\end{aligned}$$

Now, we can solve for the expression of $C_V(R)$ through Equation 8,

$$\begin{aligned}
C_V(R) &= \sqrt{\frac{\mathbb{E}(1/T)}{\lambda} - 1} \\
&= \sqrt{\frac{ae^{a\tau}\Gamma(0, a\tau)(1 + a\tau)}{a} - 1} \\
&= \sqrt{(1 + a\tau)e^{a\tau}\Gamma(0, a\tau) - 1}.
\end{aligned}$$

5 MIXTURE OF TWO EXPONENTIAL DISTRIBUTIONS WITH REFRACTORY PERIOD

The mean $(1/\lambda)$ of the mixture of two exponential distributions with refractory period τ, and pdf given by Equation 58 is,

$$\begin{aligned}
\mathbb{E}(T) &= \int_{\tau}^{\infty} t f_T(t)\mathrm{d}t \\
&= \int_{\tau}^{\infty} t(pae^{-a(t-\tau)} + (1-p)be^{-b(t-\tau)})\mathrm{d}t \\
&= ap\int_{\tau}^{\infty} te^{-a(t-\tau)}\mathrm{d}t + (1-p)b\int_{\tau}^{\infty} te^{-b(t-\tau)}\mathrm{d}t \\
&= ap\frac{(1+a\tau)}{a^2} + (1-p)b\frac{(1+b\tau)}{b^2} \\
&= \frac{pb(1+a\tau)+(1-p)b(1+b\tau)}{ab},
\end{aligned}$$

Consequently, we get Equation 59,

$$\lambda = \frac{ab}{pb(1+a\tau)+(1-p)a(1+b\tau)}.$$

REFERENCES

DLMF (2020). *NIST Digital Library of Mathematical Functions* F. W. J. Olver, A. B. Olde Daalhuis, D. W. Lozier, B. I. Schneider, R. F. Boisvert, C. W. Clark, B. R. Miller, B. V. Saunders, H. S. Cohl, and M. A. McClain, eds, `http://dlmf.nist.gov/`, Release 1.0.28 of 2020-09-15.

Weisstein, E. W. (2020). "Gaussian Integral." From MathWorld–A Wolfram Web Resource, `https://mathworld.wolfram.com/GaussianIntegral.html`

Wolfram Research, Inc. (2020). Mathematica, Version 12.0, Champaign, IL, `https://www.wolfram.com/mathematica`
